# The Prevalence of Hearing Impairments in Women with Silicone Breast Implants

**DOI:** 10.3390/diseases11010031

**Published:** 2023-02-09

**Authors:** Assaf Greenbaum, Gilad Halpert, Arad Dotan, Shaked Shivatzki, Harald Heidecke, Ricky Kaplan Neeman, Michael Ehrenfeld, Amit Wolfovitz, Howard Amital, Yael Henkin, Yehuda Shoenfeld

**Affiliations:** 1 Hadassah Medical School, The Hebrew University, Jerusalem 9190401, Israel; 2 Zabludowicz Center for Autoimmune Diseases, Sheba Medical Center, Tel-Hashomer 5262000, Israel; 3 Sackler Faculty of Medicine, Tel Aviv University, Tel Aviv 6195001, Israel; 4 Department of Otolaryngology and Head & Neck Surgery, Sheba Medical Center, Tel-Hashomer 5262000, Israel; 5 CellTrend GmbH, 14943 Luckenwalde, Germany; 6 Hearing, Speech, and Language Center, Sheba Medical Center, Tel-Hashomer 5262000, Israel; 7 Department of Communication Disorders, Sackler Faculty of Medicine, Tel Aviv University, Tel Aviv 6195001, Israel; 8 Department of Medicine ‘B’, Sheba Medical Center, Tel-Hashomer 5262000, Israel

**Keywords:** silicone breast implants, hearing impairments, tinnitus, autoimmune/inflammatory syndrome induced by adjuvants (ASIA), autoimmunity, autonomic nervous system

## Abstract

Many women with silicone breast implants (SBIs) report non-specific complaints, including hearing impairments. Hearing impairment appears to be associated with a number of autoimmune conditions. The current study aimed to evaluate the prevalence and severity of hearing impairments among women with SBIs and to explore potential improvements in their hearing capability following implant removal. Symptomatic women with SBIs (*n* = 160) underwent an initial anamnestic interview, and women who reported hearing impairments were selected for the study. These women completed self-report telephone questionnaires regarding their hearing difficulties. Some of these women underwent subjective and objective hearing tests. Out of 159 (50.3%) symptomatic women with SBIs, 80 reported hearing impairments, including hearing loss (44/80; 55%) and tinnitus (45/80; 56.2%). Five out of seven (71.4%) women who underwent an audiologic evaluation exhibited hearing loss. Of women who underwent silicone implant removal, 27 out of 47 (57.4%) reported the improvement or resolution of their hearing complaints. In conclusion, hearing impairment is a frequent complaint among symptomatic women with SBIs, and tinnitus was found to be the most common complaint. A significant reduction in hearing difficulties was observed following silicone implant removal. Further studies using larger populations are needed to verify the occurrence of hearing impairments in these women.

## 1. Introduction

Silicone is a polymeric compound comprising a silicon–oxygen unit with two organic groups attached to it, thus forming rubber-like materials, which have been used for many medical purposes, such as breast implants, joint implants, testicular prostheses, intraocular implants, artificial cardiac valves, cosmetic rhinoplasty, various shunts and catheters [1].

Silicone breast implants (SBIs) are commonly used for cosmetic purposes or in women requiring breast reconstruction in cases of breast malignancy. The cosmetic use of silicone breast implants was first introduced in 1962. This procedure became extremely popular among women all over the world, and to date, millions of women have undergone silicone breast implantation for cosmetic purposes [1]. Silicone was originally believed to be a biologically inert material; however, it is now clear that the silicone “microparticle” can migrate to local or distant sites outside the ruptured capsule of the breast implant and has been demonstrated to be able to migrate through an intact capsule, a so-called “gel bleed” [2]. In recent years, there has been a debate about the safety of silicone implants, with two main issues of concern—the induction of autoimmunity and lymphomas [3,4,5,6,7,8,9,10]. A few diagnoses, such as “human adjuvant disease” [11], “autoimmune/inflammatory syndrome induced by adjuvants (ASIA)” [12] and “silicone induced human adjuvant/autoimmune disease” [13], have been used to describe the detrimental effects of silicone and other adjuvants on the immune system.

In addition, after several reports of breast-implant-associated anaplastic large-cell lymphoma [14], the US Food and Drug Administration (FDA) recommended a boxed warning statement for silicone implants that indicates the risks associated with silicone implants. Complications that were mentioned included chronic fatigue, joint pain and a rare type of cancer.

The connection that links SBIs and autoimmunity may be supported by the concept of autoimmune/inflammatory syndrome induced by adjuvants (Shoenfeld’s ASIA syndrome), which includes several autoimmune conditions that are induced following exposure to substances with adjuvant activity [12].

In a recent large-scale study [15] of 24,651 SBI recipients compared to 98,604 non-silicone-implanted women, the authors demonstrated a positive link between silicone breast implants and the presence of autoimmune/rheumatic diseases. The disorders that were most strongly associated with SBIs included Sjogren’s syndrome, systemic sclerosis and sarcoidosis. The hazard ratio for at least one autoimmune/rheumatic disorder among women with SBIs was 1.45 (95% confidence interval 1.21–1.73) as compared to women without SBIs.

Furthermore, a previous study [16] reported a heightened production of a variety of classical autoimmune autoantibodies in both women with and without symptoms who had silicone breast implants, lending support to the connection between SBIs and autoimmunity.

Recently, we studied a large group of women with SBIs who reported various non-specific systemic symptoms, such as chronic fatigue, sleep and memory impairments, myalgia, arthralgia, dry mouth and eye and many more. These symptoms have been previously reported in women with SBIs [17,18]. Hearing impairment was one of the most common symptoms among this group of women. Their subjective complaints were mainly hearing loss and tinnitus.

Sensorineural hearing loss (SNHL) is caused by damage to the structures in the inner ear, the auditory nerve, and/or auditory pathways in the central nervous system. SNHL might be genetic or acquired and includes a variety of hearing impairments. Autoimmune inner ear disease is a rare condition that has been defined as progressive, fluctuating unilateral or bilateral SNHL. Its pathogenesis has not been elucidated [19,20]. This condition was first described by McCabe in 1979, who suggested that it was autoimmune in origin, as patients typically benefited from steroid therapy. It is worth mentioning that hearing impairments were found to be reported in suspected immunological/autoimmune-related disorders such as fibromyalgia [21,22] and COVID-19 [23,24,25,26], where some of the patients suffered from similar subjective, autonomic and unexplained symptoms to those in patients with silicone breast illness.

Tinnitus, the perception of sound in the absence of an external auditory stimulus, may be related to many factors. The sound sensation may be ongoing or pulsatile in its pattern. Tinnitus can be classified as objective or subjective, given the patient’s condition. Tinnitus will be classified as objective if the sound is generated from the patient’s own body and is audible to an examiner. However, subjective tinnitus is much more common, but in this case, there is no inner sound produced by the body but a subjective self-reported sensation [27].

In addition, patients with systemic autoimmune diseases may experience tinnitus as well as vestibular symptoms and muffled hearing [28]. Autoimmunity appears to be associated with tinnitus in a number of autoimmune conditions that are often related to hearing loss, including vasculitis, Sjogren’s syndrome, ankylosing spondylitis and systemic lupus erythematosus (SLE) [29,30,31,32].

Tinnitus is a common disorder among adults worldwide, with an estimated prevalence of 10–15% in the US [33] and with a similar percentage in European countries [34,35], Japan [36] and Korea [37]. The data usually indicate that the prevalence of tinnitus in men is higher than in women [34,36,38] and increases with age, reaching a peak in the 60–69 age group [33].

In addition, although it is a common disorder, it has been shown in the past that there is a link between tinnitus and impaired quality of life as well as symptoms of depression, anxiety and sleep disorders [39,40]. This impairment in quality of life proves the importance of recognizing and treating this disorder.

In the present study, we aimed to examine whether there is an increase in the incidence of subjective complaints of hearing impairment, in addition to objective tests in SBI women. Moreover, we assessed whether an improvement in hearing impairment was noted following silicone implant removal.

## 2. Material and Methods

### 2.1. Subjects

Participating women were from all over the State of Israel and complained of non-specific symptoms that appeared in association with silicone breast implants over time. In recent years, the awareness of autoimmune symptoms associated with women with silicone breast implants has increased, and accordingly, women suffering from these clinical manifestations have contacted the Zabludowicz Center for Autoimmune Diseases at the Sheba Medical Center. The inclusion criterion was symptomatic women with a history of breast augmentation surgery (for cosmetic or reconstructive purposes). The exclusion criteria included the removal of SBIs before the initial appointment with the physician at our center.

The study population included 160 symptomatic women with silicone breast implants who came to our institute during the years 2019–2021. These women underwent a comprehensive anamnestic questionnaire indicating the diverse systemic symptoms they suffered from, such as fatigue, muscle pain, memory impairment and sleep disturbances, as well as hearing loss, muffled hearing and tinnitus. Among the 160 women, there were women who underwent surgery for cosmetic reasons and for reasons of reconstruction following resection of breast cancer. Their age range was 22–75 with an average age of 43.7 (±10.48).

### 2.2. Data Collection

The 160 SBI women who were studied underwent a comprehensive anamnestic questionnaire that included demographic details, previous illnesses and therapy, and family history, as well as the current illness, including the symptoms and time frame of their appearance. The non-specific complaints were numerous and mostly fatigue, muscle and joint pain, memory impairment and sleep disturbances. In addition, among the symptoms the women complained of were hearing loss and tinnitus. The questionnaire also included information about the reason for implantation and time since implantation. Women who complained of hearing loss, muffled hearing and tinnitus provided details regarding their hearing impairments by means of a telephone questionnaire. The phone questionnaire was used due to the COVID-19 pandemic and difficulties meeting the SBI women personally.

### 2.3. The Questionnaire

The questionnaire included two topics. First, there were questions about hearing loss or hearing impairment complaints, such as the self-definition of their hearing sensitivity. The SBI women who complained of tinnitus in the initial interview were asked about their tinnitus characteristics, including the level of nuisance and loudness. In women who underwent implant removal, a further interview concerning improvements in their hearing impairments was conducted.

### 2.4. Hearing Evaluation

A comprehensive hearing evaluation was performed in seven women who complained of hearing impairments and agreed to be tested. The hearing evaluation included the following: audiometry, a word recognition test, tympanometry, acoustic reflex, transient evoked otoacoustic emissions (TEOAE) and auditory brainstem response (ABR) testing.

### 2.5. Statistical Analysis

Continuous variables are presented as means. Ordinal variables are presented as medians. Categorical variables are presented as counts (%). All tests used were two-tailed, and *p* < 0.05 was considered statistically significant. Statistical analysis was performed using the IBM SPSS Statistics for windows (Version 23.0) software platform.

We included several conditions as “hearing impairment” in this study, including hearing loss, muffled hearing, tinnitus and others. Moreover, we categorized hearing impairment into ordinal variables (1–4) based on the hearing impairments reported. The Wilcoxon signed-rank test was used to determine the effect of SBI removal by comparing the number of hearing impairments before and after the removal of SBIs.

## 3. Results

The mean age of the 160 women was 43.7 (±10.48) years. SBIs were implanted for cosmetic reasons in 128/159 women (80.5%) and for reconstruction purposes in 31/159 women (19.5%). The mean time from SBI implantation to symptom onset was 10.6 (±5.9) years. Of 158 (53.8%) women, 85 underwent the removal of their silicone implants.

Of the 159 women, 80 reported hearing impairments (50.3%). For one woman, we were unable to obtain this information.

**Figure 1:**
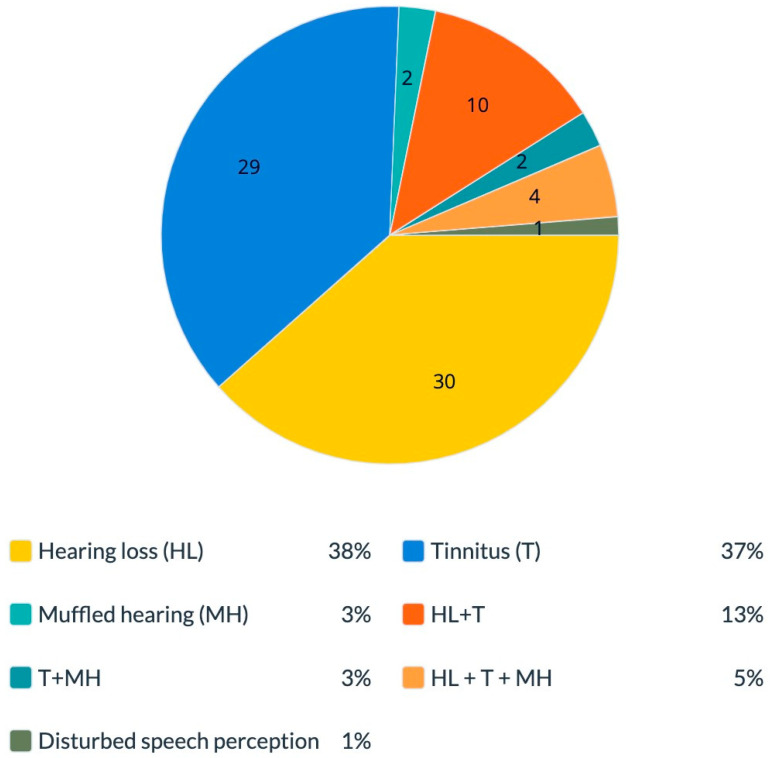
Hearing manifestations in women with silicone breast implants. The percentage of different hearing complaints among symptomatic women who complained of hearing impairments. Total 100% is equal to *n* = 78 subjects. HL—hearing loss; T—tinnitus; MH—muffled hearing.

Classified by complaints, we found that out of the women who complained of hearing impairments, 30 (38.4%) reported hearing loss, and 29 (37.1%) reported tinnitus. Furthermore, 2 (2.5%) reported muffled hearing, 10 (12.8%) reported hearing loss combined with tinnitus, 2 (2.5%) reported tinnitus combined with muffled hearing, 4 (5.1%) reported a combination of the three complaints (hearing loss, tinnitus and muffled hearing), and 1 (1.2%) reported “disturbed speech perception with multiple speakers”. For two women, we received no further information other than “hearing impairment” (Figure 1).

As noted above, 85 patients underwent implant removal. Out of the 80 women who reported hearing impairments, 52 underwent implant removal (65%).

Of the 44 (61.3%) women who complained of hearing loss, 27 underwent the surgical removal of their implants. Out of the 27 women whose implants had been removed, 9 (33.3%) reported improvement in or recovery from hearing loss, and 16 (59.2%) reported a lack of improvement. For two (7.4%) women, we were unable to obtain this information.

Of the 45 (71.1%) women who complained of tinnitus, 32 underwent the surgical removal of their implants. Out of the 32 women who removed their implants, 22 (68.7%) reported improvement in or recovery from tinnitus, and 9 (28.1%) reported a lack of improvement. For one (3.1%) woman, we were unable to obtain this information.

Six of the eight (75%) women who complained of muffled hearing underwent the surgical removal of their implants. Out of the six women who removed their implants, three (50%) reported improvement or recovery, and three (50%) reported a lack of improvement.

**Figure 2:**
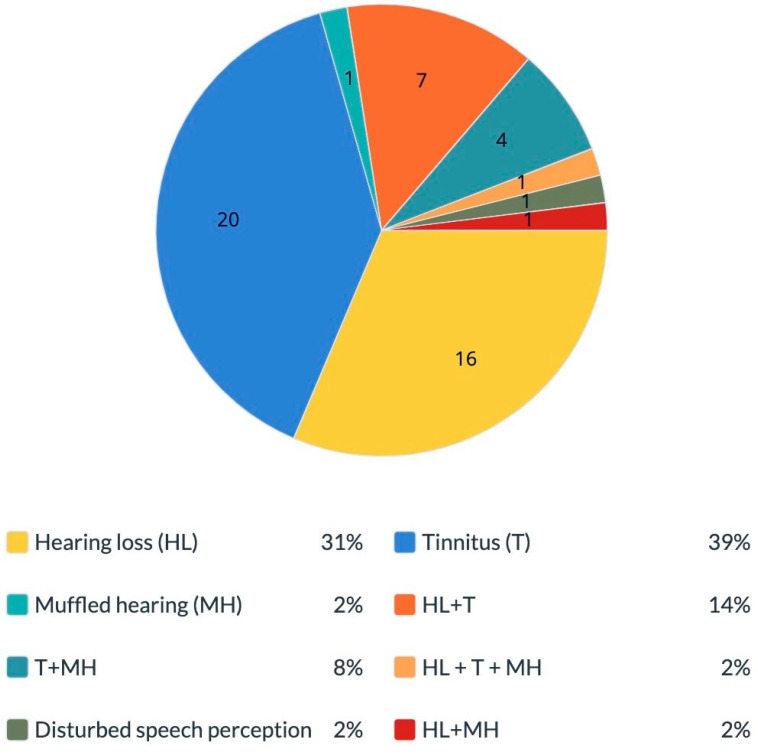
Hearing manifestations before silicone breast implant removal among women who underwent implant removal. The percentage of different hearing complaints among symptomatic women who complained of hearing impairments before they underwent silicone implant removal surgery. Total 100% is equal to *n* = 51 subjects. HL—hearing loss; T—tinnitus; MH—muffled hearing.

We focused on the group of women who complained of hearing impairments and also underwent implant removal surgery and compared the reports before and after implant removal. We found that before silicone breast implant removal, 16 (32%) reported hearing loss and 20 (40%) reported tinnitus. In addition, one (2%) reported muffled hearing, seven (14%) reported hearing loss combined with tinnitus, one (2%) reported tinnitus combined with muffled hearing, four (8%) reported a combination of the three complaints (hearing loss, tinnitus and muffled hearing) and one (2%) had “disturbed speech perception with multiple speakers” (Figure 2).

**Figure 3:**
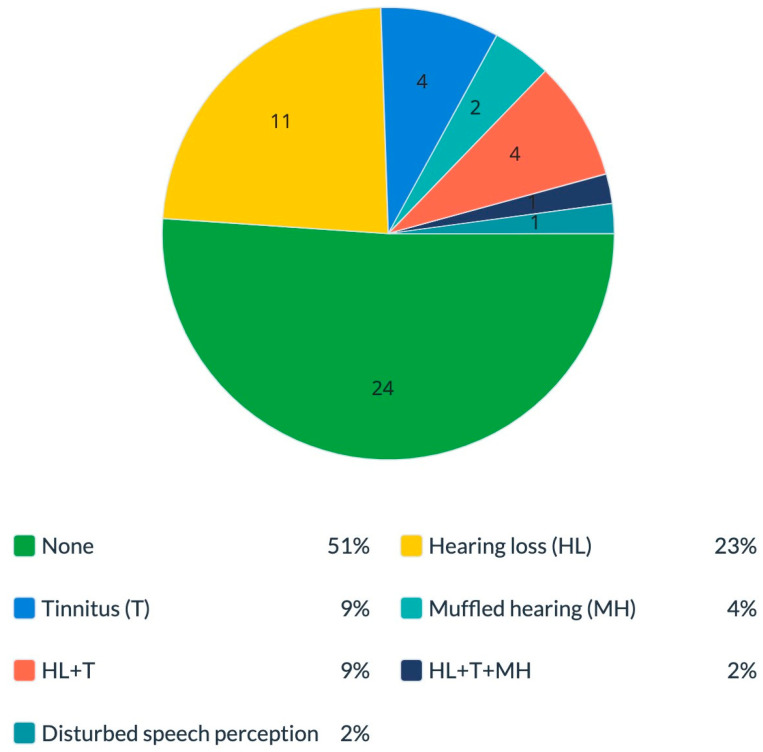
Hearing manifestations after silicone breast implant removal among women who underwent implant removal. The percentage of different hearing manifestations among symptomatic women who complained of hearing impairments after they underwent silicone implant removal surgery. Total 100% is equal to *n* = 47 subjects. HL—hearing loss; T—tinnitus; MH—muffled hearing.

When we checked the women’s reports after they underwent silicone breast implant removal, we found that 24 (51%) reported no symptoms, 11 (23%) reported hearing loss and 4 (8.5%) reported tinnitus. Furthermore, two (4.2%) reported muffled hearing, four (8.5%) reported hearing loss combined with tinnitus, one (2.1%) reported a combination of the three complaints (hearing loss, tinnitus and muffled hearing) and one (2.1%) had “disturbed speech perception with multiple speakers” (Figure 3).

**Figure 4:**
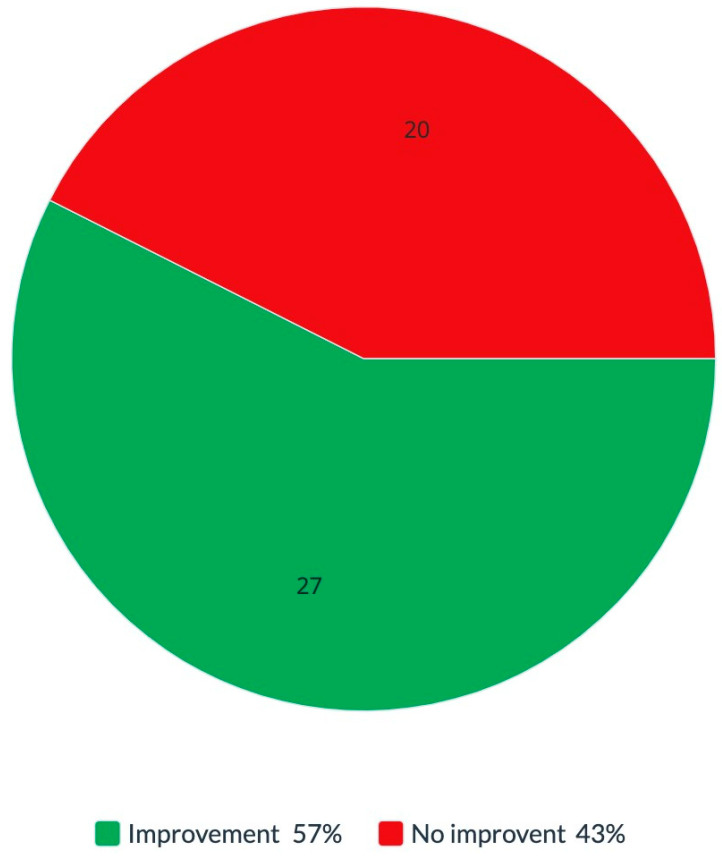
Hearing improvement after silicone breast implant removal. The percentage of symptom improvements after silicone breast implant removal. Total 100% is equal to *n* = 47 subjects.

Among the women who reported hearing impairments and underwent implant removal, 47 (90.3%) of them reported whether a change occurred in their hearing impairments following removal. Out of the 47 women who reported changes, 27 (57.4%) reported improvement in or recovery from their hearing impairments, while 20 (42.6%) reported no change (Figure 4).

Our findings indicate that reported conditions of SBI-related hearing impairments significantly improve after the removal of SBIs (*n* = 47; Z = −4.863; *p* value < 0.0001).

The most common hearing impairment reported by SBI women was tinnitus, which was reported by 45/80 (56.2%) women. It should be noted that tinnitus was one of the most prevalent complaints among the 160 women with SBIs.

As for the hearing evaluation (Table 1), five out of seven (71.4%) women who underwent an audiologic evaluation exhibited hearing loss. Of these five women, three (60%) showed abnormal acoustic reflex thresholds, and two of the three also exhibited abnormal auditory brainstem response (ABR) results. One additional woman exhibited abnormal acoustic reflex results in the presence of hearing within the normal range (based on audiometry), yet she complained of hearing loss and tinnitus.

## 4. Discussion

Our retrospective cohort study aimed to evaluate the prevalence of hearing impairments associated with silicone breast implants as part of the ASIA syndrome. Our data showed an increase in self-reported hearing impairments, including hearing loss, muffled hearing and tinnitus among our cohort. Importantly, we found that the removal of SBI resulted in an improvement in hearing impairment. Interestingly, Kim et al. [41] could not find a significant effect of implant removal regarding the improvement of hearing abnormalities.

The results of our study included a few major findings: 50.3% of the woman who arrived at our clinic because they were symptomatic and suffering from clinical manifestations reported hearing abnormalities. Of the women who underwent implant removal surgery, 57.4% reported improvements in or recovery from hearing abnormalities. Focusing on tinnitus, the improvement was greater: 68.7% reported improvement in or recovery from tinnitus after removing their silicone implants.

Tinnitus is a slightly confusing symptom, which usually makes it difficult to diagnose. Subjective tinnitus can only be heard by the person suffering from it. Currently, there are no objective diagnostic tools available to make a definite diagnosis of tinnitus. The effects of tinnitus can be assessed through several health questionnaires [42]. Therefore, we used a questionnaire that requires a self-report on the loudness and nuisance of tinnitus. In order to create a questionnaire that would be accessible to a diverse population and easy to answer, we created our own unique questionnaire, which is not included as one of the validated questionnaires that are currently used for diagnosing tinnitus.

As shown in Figure 3 and Figure 4, around 57% of women who decided to remove their implants reported a significant improvement in or even full recovery from their initial hearing impairments. Literature reviews describing recovery from tinnitus with either intratympanic injection of placebo gel [43] or stapedotomy [44] demonstrated some subjective tinnitus improvement in patients with otosclerosis. Furthermore, there is also evidence of the improvement and resolution of ASIA syndrome symptoms following the removal of the adjuvant that was implicated in this syndrome [45]. Moreover, in a recent review, Cohen Tervaert et al. [46] noted that the removal of implants is the most successful treatment for SBI symptoms and will resolve symptoms in most women. There is a possibility that this improvement in symptoms appeared due to implant removal since our results point to the relief of tinnitus in women who removed their implants. If the symptoms were not caused by SBIs, they would have not been expected to improve spontaneously after silicone implant removal. Notably, the fact that most of the symptomatic women with SBIs reported a predictable cluster of common symptoms strengthens our hypothesis that the underlying mechanism of silicone breast illness may be supported by the ASIA syndrome [47,48]. Another essential point to note is that, in our study, the removal of silicone implants was performed only in some of the women and at different times during the study period. Some patients had a more extended period from implant removal to answering the questionnaire. Therefore, there is a possibility that during a longer follow-up, the patients may further improve their tinnitus condition and may even recover completely.

Seven women who reported hearing loss underwent a comprehensive audiologic evaluation. Based on audiometry, hearing loss was evident in five of the seven women, most of whom exhibited high-frequency sensory neural hearing loss. This finding is in keeping with the results of a recent review on audiologic manifestations of autoimmune inner ear diseases [28]. Integrating the results of the audiologic test battery performed in the current study, including tympanometry, acoustic reflex, TEOAE and ABR, suggests that auditory impairments were of mixed origin, i.e., cochlear and retrocochlear sites of lesions. Specifically, three of the five women with hearing loss exhibited (1) the impaired function of outer hair cells (based on TEOAE results) and/or absent wave I of the ABR, supporting a cochlear lesion, and (2) absent or elevated acoustic reflex thresholds and/or prolonged latencies of waves III and V of the ABR, supporting a retrocochlear lesion. Evidence of a retrocochlear lesion was found in one woman. An additional woman showed results within the normal range, yet she exhibited mild hearing loss and reported tinnitus. While there are scarce data regarding audiologic manifestations of autoimmune inner ear disease, it is thought to reflect cochlear damage [49]. Interestingly, the current small cohort provides evidence of a combination of cochlear and retrocochlear lesions, and further investigation in a larger sample of patients is required.

It is worth mentioning that, as detailed in Table 1, five of the seven women indeed exhibited hearing loss that was not age-appropriate. This interpretation is based on normative data from a very large cohort of 40,728 women who were divided into age groups by decade from 20 to 89 years from Norway (Engdahl et al., 2005) [50]. For the majority of women included in the current study (73/80), we obtained self-report data only that clearly indicated that this group of patients reported hearing symptoms for the first time. Based on a large cohort study [50], women younger than 49 years are not expected to exhibit hearing loss (thresholds greater than 25dBHL at 500–6000 Hz (Hoffman et al., 2017)) [51]. Thus, 80% of our cohort (58/73) for which we had self-report data were younger than 49 years and not expected to exhibit hearing loss.

While providing valuable novel knowledge about hearing abnormalities, including hearing loss and tinnitus, among SBI women, this study has several limitations. One of them is the assessment of hearing abnormality complaints only in a selected group of patients who approached our institute with complaints regarding their medical conditions. This assessment may not reflect the group of SBI women suffering from hearing loss and tinnitus. Moreover, a pre-morbid evaluation was not available; thus, prior hearing loss cannot be ruled out. Finally, a larger group of SBI women should be studied prospectively in order to better characterize hearing impairments.

## 5. Conclusions

In this study, we found that hearing impairments are commonly reported by symptomatic SBI women. Tinnitus was the most common hearing complaint reported by these women. Using questionnaires and reports collected before and after implant removal surgery, we found that there was a significant improvement in symptoms, with the most notable improvement in self-reported tinnitus. These findings suggest that hearing abnormalities, which include hearing loss, muffled hearing and tinnitus, are causally related to silicone breast implants. The connection between SBI and hearing impairments can be explained by the ASIA syndrome and the “adjuvant hypothesis”, and therefore, the removal of silicone implants may resolve these symptoms. Further studies using larger populations of women with SBIs are needed in order to verify the occurrence of hearing impairments in these women.

## Figures and Tables

**Table 1:** Hearing evaluation via audiometry, self-report regarding hearing function, word recognition scores, tympanometry, acoustic reflex, TEOAE and ABR in 7 women with SBIs. HL = hearing loss; Rt = right; Lt = left; HTL = high-tone loss; TEOAE = transient evoked otoacoustic emissions; ABR = auditory brainstem response. #—Consecutive numbers of subjects.

Patient # Age Self-Report	Hearing Loss	Word Recognition Score (Normal Range: 88–100%)	Tympanometry	Acoustic Reflex: Ipsilateral Thresholds at 0.5, 1, 2 kHz (Normal Range: 70–100 dBHL)	TEOAE at 1, 1.5, 2, 3, 4 kHz	ABR Morphology Latencies and Brainstem Transmission Time
#1, 57 years Bilateral HL	Bilateral, mild to severe HTL	Within normal range	Type A	Rt: Elevated or no response Lt: Elevated or no response	Present bilaterally at 1, 1.5 kHz	Bilateral absence of wave I Rt: prolonged latencies of waves III, V Lt: latencies within the normal range
#2, 32 years Bilateral tinnitus	No	Within normal range	Type A	Rt: Normal Lt: Normal	Present bilaterally at all frequencies	Rt: Normal Lt: Normal
#3, 31 years Bilateral HL and tinnitus	No	Within normal range	Type A	Rt: Elevated or no response Lt: Elevated	Present bilaterally at all frequencies	Rt: Normal Lt: Normal
#4, 49 years Bilateral HL and tinnitus	Bilateral, moderate HTL	Within normal range	Type A	Rt: Elevated or no response Lt: Elevated or no response	Present bilaterally at 1–3 kHz	Bilateral absence of wave I Rt: latencies within the normal range Lt: latencies within the normal range
#5, 43 years Bilateral HL	Bilateral, notched at 2 kHz, Lt > Rt	Within normal range	Type A	Rt: No response Lt: Normal	Rt: present at all frequencies Lt: present at 1, 1.5, 4 kHz	Rt: Normal Lt: Normal
#6, 46 years No HL, difficulty understanding speech	Bilateral, mild HTL	Within normal range	Type A	Normal	Rt: present at 1.5–4 kHz Lt: present at all frequencies	Rt: Normal Lt: Normal
#7, 49 years Bilateral HL and tinnitus (Rt > Lt)	Bilateral, mild HTL	Within normal range	Rt: Type AD Lt: Type A	Normal	Present bilaterally at 1.5–4 kHz	Rt: Normal Lt: Normal

## Data Availability

The data presented in this study are available on request from the corresponding author. The data are not publicly available due to ethical restriction.

## Abbreviations

SBIs: silicone breast implants; ASIA: autoimmune/inflammatory syndrome induced by adjuvants; SNHL; sensorineural hearing loss.
